# Identification of Sarcosine as a Target Molecule for the Canine Olfactory Detection of Prostate Carcinoma

**DOI:** 10.1038/s41598-018-23072-4

**Published:** 2018-03-21

**Authors:** Dalibor Pacik, Mariana Plevova, Lucie Urbanova, Zuzana Lackova, Vladislav Strmiska, Alois Necas, Zbynek Heger, Vojtech Adam

**Affiliations:** 1 Department of Urology, University Hospital Brno, Jihlavska 20, Brno, CZ-625 00 Czech Republic; 2 Small Animal Clinic, Faculty of Veterinary Medicine, University of Veterinary and Pharmaceutical Sciences Brno, Palackeho 1, CZ-612 42 Brno, Czech Republic; 3 Department of Chemistry and Biochemistry, Mendel University in Brno, Zemedelska 1, CZ-613 00 Brno, Czech Republic; 4 Central European Institute of Technology, Brno University of Technology, Technicka 3058/10, CZ-616 00 Brno, Czech Republic; 5 Faculty of Medicine, Masaryk University, Kamenice 5, CZ-625 00 Brno, Czech Republic

## Abstract

The hypothesis that dogs can detect malignant tumours through the identification of specific molecules is nearly 30 years old. To date, several reports have described the successful detection of distinct types of cancer. However, is still a lack of data regarding the specific molecules that can be recognized by a dog’s olfactory apparatus. Hence, we performed a study with artificially prepared, well-characterized urinary specimens that were enriched with sarcosine, a widely reported urinary biomarker for prostate cancer (PCa). For the purposes of the study, a German shepherd dog was utilized for analyses of 60 positive and 120 negative samples. Our study provides the first evidence that a sniffer dog specially trained for the olfactory detection of PCa can recognize sarcosine in artificial urine with a performance [sensitivity of 90%, specificity of 95%, and precision of 90% for the highest amount of sarcosine (10 µmol/L)] that is comparable to the identification of PCa-diagnosed subjects (sensitivity of 93.5% and specificity of 91.6%). This study casts light on the unrevealed phenomenon of PCa olfactory detection and opens the door for further studies with canine olfactory detection and cancer diagnostics.

## Introduction

Prostate cancer (PCa) remains the second most frequent malignant disease in men and the third cause of death among men in the US and Western Europe, with 1.1 million newly diagnosed cases in 2012, representing 15% of all oncological diagnoses in men^1^. Although it has been almost 30 years from establishing the prostate-specific antigen (PSA) in clinical practice, PCa diagnostics and screening remain inefficient and rely on the quantitation of PSA combined with a digital rectal examination (DRE). Hence, substantial effort is being put into discovering new approaches for PCa detection, which ideally should be sufficiently reliable, PCa-specific and sensitive for early-stage detection^2^.

In our previous study, we demonstrated that a specially trained sniffer dog can reach high sensitivity (93.5%) and specificity (91.6%) in diagnosing histologically confirmed PCa using its olfactory abilities by sniffing urinary specimens from patients with PCa^3^. The hypothesis that dogs may be able to detect malignant tumours on the basis of odour is not new and was first put forward by Williams and Pembroke in 1989^4^. From that time, several reports have been published describing the olfactory identification of cancers of the bladder^5^, breast and lung^6^, skin^7^ or prostate^3^.

Despite these promising results, to our knowledge, there is no information regarding the substances that are responsible for canine olfactory recognition. The most investigated substances are volatile organic compounds (VOCs), which can be produced due to tumour metabolic shifts^8^. Heterogeneous groups of VOCs can be then detected in sweat, breath, urine or stool, and these chemical groups include acids, alcohols, ketones, aldehydes, amines and others^9^.

We hypothesized that the olfactory detection of PCa based on the sniffing of urinary specimens should be enabled by the presence of sarcosine. Sarcosine (*N*-methylglycine) is a reported biomarker for PCa, of which an increased urinary level and potential for non-invasive detection of early-stage PCa was delineated in 2009 by Sreekumar *et al*.^10^. If the olfactory detection of PCa depends on the amount of sarcosine in a complex mixture of urinary compounds, then a trained dog should be able to recognize the artificial urine with the addition of sarcosine. Hence, the aim of the present study is to perform olfactory analyses of artificially prepared urinary specimens containing distinct, physiologically relevant concentrations of sarcosine with the same dog that has an excellent accuracy for PCa diagnostics, per our previously published study^3^.

## Results

In three stages from August 2016 to May 2017, the dog gradually carried out olfactory analyses of 60 positive and 120 negative samples. The stages differed only in the amount of added sarcosine in the positive samples, while the composition of the negative samples remained constant throughout all stages. Table 1 illustrates the overview of the grades of olfactory identification of positive samples across all stages. Table 2 demonstrates the classification of all olfactory analysis results, which were subsequently employed for statistical processing.

**Table 1:** Identification grading of positive samples (with added sarcosine) in three stages.

Grade	1^st^ stage	2^nd^ stage	3^rd^ stage
1	8	6	10
2	9	8	8
3	3	6	2

**Table 2:** Classification of the sarcosine olfactory detection results. TP - true positive, FP - false positive, FN - false negative, TN - true negative.

	1^st^ stage	2^nd^ stage	3^rd^ stage
TP	17	14	18
FP	3	6	2
FN	3	6	2
TN	37	34	38

In the first stage, when the amount of sarcosine in the artificial urine was 1 µmol/L, the sensitivity of olfactory detection was 85% with 92.5% specificity. In the second stage, when the concentration of added sarcosine decreased to 0.1 µmol/L, the olfactory analysis reached a sensitivity of 70% and a specificity of 85%. Finally, in the third stage with the highest amount of added sarcosine (10.0 µmol/L), we identified the highest sensitivity (90%) and specificity (95%), underpinning the importance of sarcosine in the success rate of olfactory analyses.

Using these results, the predictive value of the positive test (or precision) is 90% with an LR of 18 for positive samples and 10.5 for negative samples. These values show the exceptional accuracy of the sniffer dog and demonstrate that sarcosine could be one of the pivotal urinary molecules that are recognized by a dog’s olfactory abilities. Importantly, we also verified that sarcosine concentration is not decreased during olfactory analysis due to biochemical instability and degradation (Table 3).

**Table 3:** IEC-Vis verification of sarcosine stability before and after olfactory analyses.

Sarcosine (µmol/L)	*n*	1^st^ stage		2^nd^ stage		3^rd^ stage	
		BOA*	AOA**	BOA	AOA	BOA	AOA
		Mean ± SD		Mean ± SD		Mean ± SD	
0.1	20	0.095 ± 0.04	0.097 ± 0.08	0.11 ± 0.06	0.099 ± 0.01	0. 10 ± 0.02	0.098 ± 0.01
1.0	20	1.01 ± 0. 16	0.97 ± 0.11	0.98 ± 0.11	1.04 ± 0.14	0.95 ± 0.08	0.97 ± 0.10
10.0	20	9.71 ± 1.26	10.06 ± 1.87	10.04 ± 2.01	9.99 ± 1.48	10.05 ± 1.99	10.10 ± 2.68

*BOA - before olfactory analysis.

**AOA - after olfactory analysis.

## Discussion

Scent is a well-developed sense in most animal species. Distinct odours are detected by olfactory receptors (ORs) expressed in the olfactory epithelium of the nasal cavity. In 2014, Niimura *et al*. delineated the numbers of orthologous genes encoding ORs, and the dog (*Canis lupus* f. *familiaris*) ranked 9^th^ place with 811 genes^11^. For comparison, 1^st^ place belongs to the African elephant (*Laxodonta africana*) with 1948 OR genes. Hence, the dog is the best choice for olfactory detection because of two key aspects: *i*) the dog is capable of intensive training, and *ii*) the dog can be easily handled while detecting very low concentrations of a target substance^12^.

In 2008, Gordon *et al*. attempted to expand the dog’s exceptional olfactory capabilities to the area of PCa detection^13^. However, due to procedural errors, the study did not bring positive results. Despite that, the publication was significant for the success of future studies. In 2011, the first successful olfactory analysis of PCa was performed by Cornu *et al*.^14^. The study demonstrated that a specially trained dog can detect PCa from urinary specimens with a sensitivity and specificity of 91%. Similar results have been achieved in our pilot study (sensitivity of 93.5% and specificity of 91.6%)^3^ and in a study by Taverna *et al*. who used two dogs and reached a sensitivity of 98.6–100% and a specificity of 97.6–98.7%. All abovementioned studies demonstrate that through intensive training, the dog’s exceptional olfactory apparatus can recognize PCa, most likely due to the presence of urinary VOCs. Despite that, to our knowledge, no one has performed an experiment with a well-characterized artificial matrix to identify the targets for olfactory detection.

Hence, the answer to the question: “What do sniffer dogs really smell in the urine?” could provide significant insight into the potential of non-invasive biomarkers for PCa laboratory diagnostics, which currently rely on PSA, which has a relatively poor discriminating ability in men with symptomatic benign prostatic hyperplasia^15^. This often results in false positivity and unnecessary biopsies. Regardless, even in 2017, there is no clue to which of the urinary compounds is responsible for a high performance of PCa olfactory detection.

Our study was performed in regard to the excellent results of our sniffer dog in detecting PCa from the 2015 study^3^ and the fact that urinary sarcosine appears to be a potential PCa biomarker, whose concentrations are increased in the urine of PCa-diagnosed patients^10,16^. Because we are using an animal as a “sensory device”, several issues should be mentioned. First, we did not carry out any training of the dog’s olfactory apparatus to identify sarcosine; hence, the dog that was trained to detect PCa could fluently follow the sniffing of artificially prepared urine. The experiments also had several pitfalls. There was a potential concern that after a new experience, the dog would not be able to continue with the detection of PCa. We also considered using sarcosine dissolved in water; however, due to concerns that a new solvent would confuse the dog trained to sniff human urine, we avoided this option. For the same reason, we avoided the use of female urine, which can confuse the dog due to the presence of oestrogen hormones. Hence, we decided to use artificial urine, which mimics human urine and its chemical composition is fully characterized. To fully imitate real urine, additions of sarcosine were selected to correspond to the values of sarcosine in urinary specimens, which have been identified in PCa-diagnosed patients^17,18^.

One advantage of our study is that we used the same dog for the detection of PCa and sarcosine. This enabled a direct comparison between sensitivity and specificity, which are significantly comparable, particularly when detecting the highest amount of sarcosine (sensitivity of 93.5% and specificity of 91.6% for PCa *vs*. 90% and 95% for sarcosine, respectively). The credibility of our results is further supported by olfactory analyses being carried out immediately after the preparation of artificial urine without freezing, which results in sarcosine degradation. Importantly, the artificial urine did not contain any enzymes that degrade sarcosine (sarcosine dehydrogenase and sarcosine oxidase) to yield glycine^16^, and thus, sarcosine remained stable throughout the olfactory detection series as shown by IEC-Vis. Notably, the achieved results may be biased due to the relatively small number of analysed specimens; however, the contribution of sarcosine to the success rate of olfactory detection is clear, as well as its dependence on sarcosine concentration.

## Materials and Methods

### Chemicals

All chemicals were purchased from Sigma-Aldrich (St. Louis, MO, USA) at American Chemical Society (ACS) purity, unless noted otherwise.

### The dog and its training

For the purposes of the study, a German shepherd dog named Agata Jankari, born on May 1, 2012, was utilized. She began a special training programme at seven months of age. Prior to that, she underwent basic obedience training and scent work training^19^. The training of the dog at a young age to utilize its olfactory abilities to diagnose PCa was based on the positive reinforcement method using a clicker as described in our previous study^3^. This method consists of marking and rewarding desired behaviours, which are indicated by a clicker and immediately rewarded using a treat or a game with a toy.

### Preparation of artificial urinary specimens with the addition of sarcosine

The urine medium was based on the analyses of constituents in common human urine, of which the midpoint values of the ranges given for each component have been used. Each batch was freshly prepared prior analysis as 0.5 L of solution containing 250 mL of distilled water (*v/v*), 1.9 g of potassium chloride (*w/v*), 4.25 g of sodium chloride (*w/v*), 12.25 g of urea (*w/v*), 0.07 g of uric acid (*w/v*), 0.52 g of magnesium sulphate heptahydrate (*w/v*), 0.52 g of citric acid (*w/v*), 0.17 g of ascorbic acid (*w/v*), 0.59 of potassium dihydrogen phosphate (*w/v*), 0.17 g of calcium chloride (*w/v*), 0.0012 g of iron(II) sulphate (*w/v*), 1.6 g of sodium sulphate (*w/v*), 0.7 g of ammonium chloride (*w/v*), 0.7 g of creatinine (*w/v*), 0.32 g of sodium hydroxide (*w/v*) (added slowly), 0.235 g of sodium bicarbonate (*w/v*), 0.14 mL of 18 M sulphuric acid (*v/v*); to add a small amounts of nucleic acids and trace elements, we added 0.005 g of yeast extract (*w/v*). Finally, 1 g of bacteriological peptone (*w/v*) was added for a mixture of amino acids. The mixture was brought to final volume (0.5 L) with water and had a pH of approximately 6.9. The resulting solution was separated into 5-mL aliquots in opaque tubes, and either non-spiked aliquots or aliquots spiked with sarcosine (0.1, 1.0 or 10.0 µmol/L) were immediately transported to the Small Animal Clinic, Faculty of Veterinary Medicine, University of Veterinary and Pharmaceutical Sciences. After 30 min of equilibration at room temperature, samples were prepared for subsequent olfactory analyses. Before and after the olfactory analyses, the amount of sarcosine in the artificial urine was verified using ion-exchange chromatography (IEC) with post-column ninhydrin derivatization and visible spectrophotometry (Vis) detection (IEC-Vis) according to our previous study^20^ to control for the possible degradation of sarcosine.

### Olfactory analysis of artificial urinary samples

The methodology was based on our previous study regarding olfactory identification of the urine of PCa-positive subjects^3^. A sniffing series always contained one positive and two negative samples randomly placed on the floor. This prevented the dog to create a link between a particular sample position and its positivity/negativity. Each series contained only three samples to fully maintain the concentration of the dog for olfactory analysis. This also allowed keeping a sufficient distance between analysed samples (at least 75 cm) to prevent fusion of odours, which could bias the analysis. Contrary to our previous study and the dog’s training, as a positive sample, laboratory-prepared artificial urine (detailed composition described above) with defined amount of sarcosine, ranging between 0.1 to 10.0 µmol/L, was used. As a negative control, we employed artificial urine without sarcosine. Samples (5 mL) were randomly positioned on the floor in uniform, opaque plastic beakers with a perforated lid. The beakers were fixed to the floor using non-perfumed adhesives (Pritt Multi Tack, Pritt, Dusseldorf, Germany) to prevent them from being knocked over. The dog handler was familiar with the position of the positive samples to reward the dog in cases of correct identification. As reward, Lakse Kronch Treat with salmon oil (Henne Pet Food, Outrup, Denmark) was used. All methods were carried out in accordance with relevant guidelines and regulations. The experimental protocols were approved by Ethical Committee of University of Veterinary and Pharmaceutical Sciences Brno, Brno, CZ.

### Grading for identification evaluation

After presenting samples to the dog, we evaluated its behaviour using three different grades as follows: grade 1, the sample was identified immediately without hesitation, *i.e*., the dog laid down by the sample while ignoring the other samples; grade 2, the sample was identified with a slight hesitation, *i.e*., the dog repeatedly sniffed it and then carried out the correct identification; grade 3, the sample was identified incorrectly, *i.e*., a false positive or false negative identification.

### Descriptive statistics

The results from the olfactory identification sessions were analysed using statistical evaluation of the accuracy of the diagnostic assay, in which the diagnostic potential of the test was validated and compared with the verifiable status of the tested object. Specifically, in the cases of behavioural grades 1 and 2, the positive results were classified as true positive (TP) and the negative results as true negative (TN). In the case of behavioural grade 3, the results were classified as false negative (FN) or false positive (FP).

The sensitivity, the probability that the analysis will give a positive result, when the sample is genuinely positive was calculated as follows:

1 $${Sensitivity}\,( \% )={TP}/({TP}+{FN}).$$

The specificity, the probability that the analysis will give a negative result, when the sample is genuinely negative was calculated as follows:

2 $$Specificity\,( \% )=TN/(FP+TN).$$

The predictive value (precision) of a positive test, the probability that a sample is positive when the olfactory analysis gives a positive result, was calculated as follows:

3 $$Predictive\,value\,of\,a\,positive\,test=TP/(TP+FP).$$

The likelihood ratio (LR) for a positive result (LR+) represents the probability ratio that the positive sample is diagnosed as a positive and the probability ratio that the negative sample is misidentified as positive, and LR+ was calculated as follows:

4 $$LR+=sensitivity/(1-specificity).$$

The LR for a negative result (LR-) represents the probability ratio that the negative sample is diagnosed as negative and the probability ratio that the positive sample is misidentified as negative, and LR- was calculated as follows:

5 $$LR-=(1-sensitivity)/specificity.$$

Generally, a high-quality diagnostic test is defined by an LR + > 10 and an LR− > 0.1.
